# Different quality of treatment in retroperitoneal sarcomas (RPS) according to hospital-case volume and surgeon-case volume: a retrospective regional analysis in Italy

**DOI:** 10.1186/s13569-018-0091-0

**Published:** 2018-02-28

**Authors:** Sergio Sandrucci, Agostino Ponzetti, Claudio Gianotti, Baudolino Mussa, Patrizia Lista, Giovanni Grignani, Marinella Mistrangelo, Oscar Bertetto, Daniela Di Cuonzo, Giovannino Ciccone

**Affiliations:** 1 Visceral Sarcoma Unit, University of Turin, Cso Dogliotti 14, 10126 Turin, Italy; 2 Medical Oncology 1 Division, Città della Salute e della Scienza, Turin, Italy; 3 Department of Surgical Sciences, University of Turin, Turin, Italy; 4 Department of Medical Oncology, Candiolo Cancer Institute-FPO, IRCCS, Candiolo, Italy; 5 Regional Oncologic Network Department, Turin, Italy; 6 Cancer Epidemiology Unit, Città della Salute e della Scienza, Turin, Italy

**Keywords:** Retroperitoneal sarcomas, Multidisciplinary management, Hospital case volume, Surgeon case volume, Quality of surgery, Retrospective analysis

## Abstract

**Background:**

Retroperitoneal sarcomas (RPS) should be surgically managed in specialized sarcoma centers. However, it is not clearly demonstrated if clinical outcome is more influenced by Center Case Volume (CCV) or by Surgeon Case Volume (SCV). The aim of this study is to retrospectively explore the relationship between CCV and SCV and the quality of surgery in a wide region of Northern Italy.

**Methods:**

We retrospectively collected data about patients M0 surgically treated for RPSs in 22 different hospitals from 2006 to 2011, dividing them in two hospital groups according to sarcoma clinical activity volume (HCV, high case volume or LCV, low case volume hospitals). The HCV group (> 100 sarcomas observed per year) included a Comprehensive Cancer Center (HVCCC) with a high sarcoma SCV (> 20 cases/year), and a Tertiary Academic Hospital (HVTCA) with multiple surgeon teams and a low sarcoma SCV (≤ 5 cases/year for each involved surgeon). All other hospitals were included in the LCV group (< 100 sarcomas observed per year).

**Results:**

Data regarding 138 patients were collected. Patients coming from LCV hospitals (66) were excluded from the analysis as prognostic data were frequently not available. Among the 72 remaining cases of HCV hospitals 60% of cases had R0/R1 margins, with a more favorable distribution of R0/R1 versus R2 in HVCCC compared to HVTCA.

**Conclusions:**

In HCV hospitals, sarcoma SCV may significantly influence RPS treatment quality. In low-volume centers surgical reports can often miss important prognostic issues and surgical quality is generally poor.

## Background

Retroperitoneal sarcomas (RPS) account for 10–15% of soft tissue sarcomas (STS) with an expected annual incidence of nearly 1500 cases in Europe and an expected 5-year overall survival (OS) of 30–35% [1]. Histopathological analysis can reveal multiple histotypes with liposarcoma and leiomyosarcoma as the most common [2].

The mainstay of treatment is surgical resection due to its survival advantage over nonsurgical treatments [3]. The intent of surgery is complete tumor resection with negative margins, which may require en bloc removal of adjacent involved organs or tissues. Of course, a wide margin per se may not be enough to guarantee an improved prognosis especially in specific histotypes (e.g. leiomyosarcoma) thus making it crucial to balance between wider excision and multimodal treatments [4]. Given the low incidence of RPS, individual hospitals and surgeons generally observe very few cases; for this reason available guidelines and consensus-papers state that, as a complex and rare disease, every case of RPS should be referred to a specialized sarcoma center and managed by a multidisciplinary team [5–7]. However, it is unclear what factor(s), for example, case volume, surgeon activity volume, hospital type, or the availability of adjuvant therapies, is/are the principal driver(s) of improved outcomes.

It is not clearly demonstrated if for STS, and specifically for RPS, clinical outcome is more influenced by center case volume (CCV) or by surgeon case volume (SCV). In the literature, the effect of surgeon versus hospital volume on outcomes after complex oncological surgery is poorly characterized [8]. Published retroperitoneal sarcoma series are mostly collected from high volume centers, in which the multidisciplinary aspect is most relevant rather than the surgeon’s caseload. The lack of surgeon-specific identifiers makes impossible to explore the interplay between hospital and surgeon volume and their impact on oncological outcomes. Therefore, it is unclear whether the principal determinant of oncological outcomes is high hospital case volume or high surgeon case volume. Providing care for RPS patients frequently requires a multidisciplinary team approach, and the team itself may be just as important than the surgeon in producing favorable outcomes.

NICE guidelines state that a surgeon with specific expertise in these tumors, who is a core member of the multidisciplinary team (MDT), is needed within a reference center; they also consider the number of new cases per year as an important quality evaluation item for sarcoma multidisciplinary teams. A sarcoma MDT should be expected to manage at least 100 new STS patients per year, and this caseload should be based either in a single hospital or in several geographically close and closely affiliated hospitals, which would constitute a sarcoma treatment network [9].

Due to the rarity of these diseases, it is difficult for a general surgeon to reach an adequate case volume. The only paper dealing with the problem of adequate surgical volumes in STS proposed a ≥ 5 sarcoma surgeries/year cut off, after an analysis of 4205 STS cases registered in the Florida Cancer Data System (FCDS) in which medical facilities above the 67th percentile for volume were defined as high-volume centers [10].

Concerning the treatment of retroperitoneal sarcomas, the aim of this study is to retrospectively explore the relationship between the hospital or surgeon case volume and the quality of surgery in a region of Northern Italy.

## Methods

We retrospectively collected data concerning two regions of northern Italy, Piedmont and Aosta Valley (with a total amount of 4.5 million of inhabitants), to identify RPS patients, without distant metastases at diagnosis, operated during the period from 2006 to 2011 in order to analyze care center characteristics (according to high or low CCV and SCV) and quality of surgical treatment. Data collection was authorized by a partnership between the Department « Rete Oncologica del Piemonte e della Valle d’Aosta » (Piedmont and Aosta Valley Oncologic Network) and Italian Pathologist Association (SIAPEC) stipulated in June 2012; all data were recorded anonymously respecting Italian privacy rules.

Data of histopathological reports from January 2006 to December 2011 were collected from local databases of 22 different hospitals. According to the type of electronic database available in every single hospital, site-specific search strings were prepared using keywords able to describe the site and the morphology (i.e. “retroperitoneum” and/or “sarcoma”) and SNOMED codes used for sarcomas morphology [11].

All extracted cases were screened by a skilled medical oncologist and collected in an encrypted database, which contained clinical and histopathological data, with particular attention to ESMO guidelines main prognostic items such as tumor size, grading, surgical margins (according to the R0, 1 and 2 ranking), preoperative biopsy and tumor integrity.

**Fig. 1:**
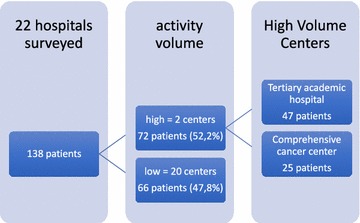
Series distribution according to activity volumes adopting the 100 cases/year cut-off rule suggested by NICE [9]

In our study patients data retrieved from different hospitals were split in two groups according to their yearly sarcoma caseload, adopting the 100 cases/year cut-off rule suggested by NICE [9] (Fig. 1).

In the “high volume” group two institutions were included:

- “Candiolo Cancer Center, a high volume Comprehensive Cancer Center (HVCCC) with nearly 150 STS cases observed per year.
- “Città della Salute e della Scienza” San Giovanni Battista hospital, a high volume Tertiary Care Academic hospital (HVTCA) with more than 100 STS cases observed per year.

In the “low volume” group all other hospitals were included (low volume secondary care hospitals, LVSCH).

In this series three different approaches to RPS are represented:

- HVCCC, a high-volume cancer center with a sarcoma-committed surgical team (high CCV and SCV > 20 surgeries per year) and a regular RPS-multidisciplinary board (RMB);
- HVTCA, a high-volume tertiary care academic hospital without a sarcoma-committed surgical team (high CCV and SCV ≤ 5 cases per year for each involved surgeon) and a formalized RMB;
- LVSCH, a group of low volume hospitals (low CCV and SCV < 5 RPS surgeries per year) without a formalized RMB.

Missing clinical informations concerning the “high volume” group were sorted from the institutional internal electronic chart database of each institution.

Missing data about the patients in charge to LVSCH were not obtained, due to the absence of a reliable database or, in case of an existing one, to access restrictions for external investigators.

Follow up was available only for the HV hospital patients; the median value was of 85 months (range 72–100).

### Statistical analysis

Data were analyzed with SAS system 9.2 software.

The crude and adjusted hazard ratios were calculated according to hospital, patient’s age, tumor size, grading, recurrent or primitive tumor. Two logistic regression models were adopted: for tumor integrity and for surgical margins (confidence limits 95%).

The Kaplan–Meier survival curve for primary/recurrent RPS was calculated on HV series. The Kaplan–Meier survival curve according to surgical margins was built with the high HCV hospitals data, and is based on 57 patients.

## Results

Data from 22 hospitals were available: 138 patients (55% males and 45% females) were identified with a diagnosis of RPS from 2006 to 2011.

According to care center volume 47 cases (34.1%) were treated in HVTCA, 25 (18.1%) in HVCCC: 66 cases (47.8%) were treated in LVSCH.

As regards this latter group of patients, the lack of essential information impaired any statistical analysis. In particular, no useful informations were available concerning tumor diameter, preoperative biopsy, margins evaluation and FNLCLCC grading. For this reason, data from this latter group was not considered in the subsequent analysis, which has been conducted only on HTVCA and HVCCC patients.

The main characteristics of this series are summarized in Table 1.

**Table 1:** patients from high volume centers (HCV) main characteristics

	Global HVC	%	HVTCA	%	HVCCC	%	HVTCA versus HVCCC
Age							
< 60	15	21.0	11	23.5	4	19.0	P = 0.81
≥ 60	57	79.0	36	76.5	21	81.0	
Sex							
M	43	59.7	24	51.0	19	76.0	P = 0.62
F	29	40.3	23	49.0	6	24.0	
Primary/recurrent							
Primary	46	63.8	32	68.0	14	56.0	P = 0.30
Recurrent	26	36.2	15	32.0	11	44.0	
Diameter							
< 10 cm	22	30.5	15	32.0	7	28.0	P = 0.26
≥ 10 cm	50	69.5	32	68.0	18	72.0	
Histotype							
Liposarcoma	40	55.5	24	51.0	16	64.0	P = ns
Leiomyosarcoma	10	14.0	9	19.0	1	4.0	
Sarcoma NOS	8	11.0	1	2.0	7	28.0	
Others	14	19.5	13	28.0	1	4.0	
Grading							
1	10	14.0	7	14.8	3	12.0	P = 0.9
2	22	31.0	18	38.2	4	19.0	
3	27	37.5	14	30.0	13	52.0	
Unknown	13	17.5	8	17.0	5	17.0	
Preoperative biopsy							
Yes	46	63.8	31	66.5	15	60.0	P = 0.45
No	26	36.2	16	33.5	10	40.0	
Margins							
R0	20	28.0	10	21.0	10	40.0	*R0* *+* *R1* versus *R2 P* *=* *0.013*
R1	23	32.0	13	28.0	10	40.0	
R2	18	25.0	15	32.0	3	12.0	
Unknown	11	15.0	9	19.0	2	8.0	
Fragmentation							
Yes	36	50.0	30	63.8	6	24.0	*P* *=* *0.01*
No	36	50.0	17	36.2	19	76.0	

Statistically significant P values are in italic

*HVTCA* high volume Tertiary Care Academic Hospital, *HVCCC* high volume Comprehensive Cancer Center

Seventeen different histotypes were observed. The most frequent was liposarcoma (55.5%), followed by leiomyosarcoma (14%), sarcoma NOS (11%) and other histotypes. The difference between the two groups was not significant.

The tumor was primitive in 63.8% and recurrent in 36.2%: in HVCCC primaries were 56% and recurrences 44%; in HVTCA 68 and 32%. (Chi Squared test, P = 0.30).

According to FNCLCC grading, 14% of tumors were G1, 31% were G2 and 37.5% G3. In 17.5% of cases, this information was not recorded. The subdivision of grades G1/G2–3 in HVCCC and HVTCA was 31/52 and 53/30 (Chi Squared test, P = 0.91), respectively.

Tumor diameter was smaller than 10 cm in 30.5% of cases (32% for HVTCA and 28% for HVCCC), greater than 10 cm in 69.5% (68% for HVTCA and 72% for HVCCC) (Chi Squared test, P = 0.2622).

A preoperative biopsy was performed in 63.8% of patients, of which 66.5% coming from HVTCA and 60% from HVCCC.

**Fig. 2:**
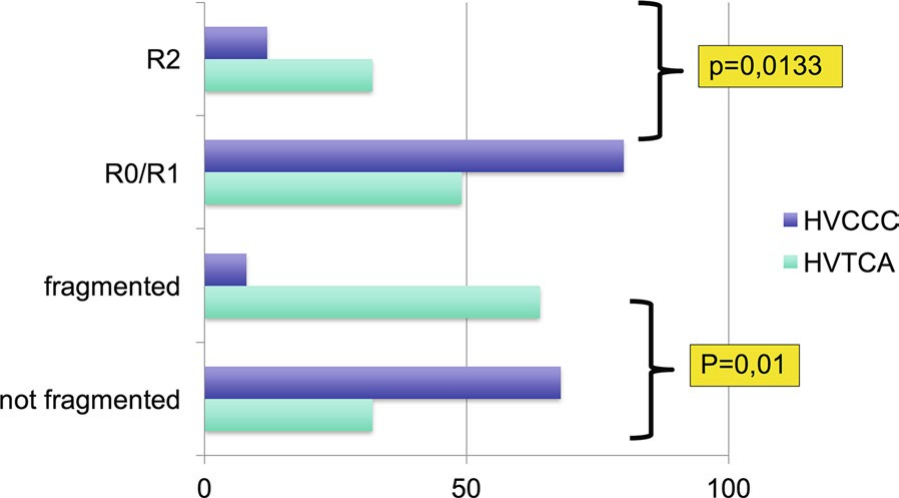
Analysis of margin involvement and specimen fragmentation according to the hospital of treatment (HVCCC versus HVTCA). P values are derived from Chi square test

According to previous experiences [12, 13] surgical resections were classified as macroscopically complete (R0 or R1) or incomplete (R2). 60% of RPS had a R0/R1 resection, 25% had R2 resection. In 15% of cases the status of surgical margins was not recorded. In HVCCC group the distribution R0/R1 versus R2 was 80 and 12%; in HVTCA, 49 and 32% (Chi Squared test, P = 0.0133; Fig. 2).

Tumors were removed intact in 50% of cases. In HVCCC group the rate of fragmented/intact specimens was 24 and 76%, and in HVTCA, 63.8 and 36.2% (Chi Squared test, P = 0.01, Fig. 2), respectively.

We compared HVTCA and HVCCC groups with the Chi squared test for grading, surgical margins, tumor size and intact specimen removal. In both logistic regression models concerning intact specimen and surgical margins (Table 2), only the “care center” item demonstrated a statistically significant correlation (i.e. HVCCC versus HVTCA) (P = 0.03, adjusted effects).

**Table 2:** Logistic regression model about the factors potentially affecting the quality of surgical margins (R0/1 versus R2)

Covariates	Rough effects	P	IC95%	Adjusted effects	P	IC95%
HVCCC^a^	–	–	–	–	–	–
HVTCA	5.262	0.0192	1.311–21.115	8.335	0.0306	1.220–57.242
Liposarcoma	–	–	–	–	–	–
Leiomyosarcoma	1.094	0.9059	0.248–4.829	1.193	0.8388	0.218–6.543
Others	1.176	0.8551	0.206–6.731	0.470	0.5620	0.037–6.034
Age	0.970	0.3390	0.912–1.032	0.973	0.4823	0.903–1.049
Primary	–	–	–	–	–	–
Recurrent	1.450	0.5490	0.430–4.889	3.252	0.1608	0.626–16.897
< 10 cm	–	–	–	–	–	–
> 10 cm	1.107	0.8830	0.285–4.297	0.808	0.8104	0.141–4.617
G1	–	–	–	–	–	–
G2/G3	0.288	0.0797	0.071–1.159	0.365	0.3485	0.045–2.999
Unknown	3.718	0.2369	0.422–32.759	1.687	0.7363	0.080–35.384

^a^*HVCCC* high volume Comprehensive Cancer Center, *HVTCA* high volume Tertiary Care Academic Hospital

**Fig. 3:**
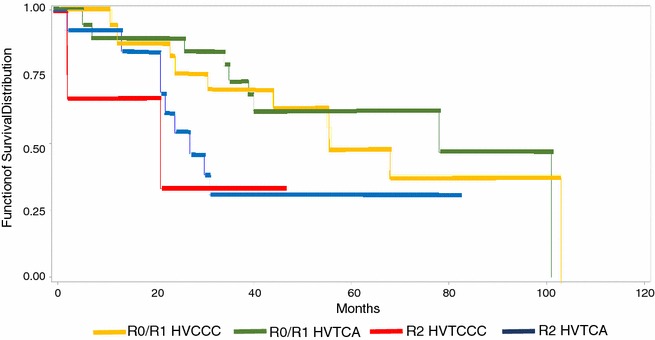
Kaplan-Meyer curves according with the status of surgical margins (R0/R1 versus R2) patients from HVCCC versus patients from HVTCA

5 years survival according to the quality of margins was 65% for R0–R1 and 31% for R2 patients (Chi Squared test, P < 0.001) without differences between HVCCC and HVTCA cases (Chi Squared test, P = 0.06 Fig. 3).

## Discussion

The outcome of surgical treatment of many common tumors (as for example rectal cancer, breast cancer, lung cancer, prostate cancer, head and neck cancers and esophageal cancer) are clearly influenced by both center case volume (CCV) and surgeon case volume (SCV) [14, 15].

In STS, several studies state that HCV hospital may assure higher survival rate [10, 16].

Some retrospective data show that the management of RPS in sarcoma-specialized centers is associated with a lower loco-regional relapse rate and a 5-year OS of nearly 60–65% [17, 18] and that high-volume centers perform surgery more adherently to clinical STS guidelines than low-volume ones [19, 20].

In real life, up to 63% of STS in UK are referred to non-specialized centers [16]; up to 50% of non-oncology committed surgeons perform extremity soft tissue sarcoma resections in California [21]. In a recent survey the German Interdisciplinary Sarcoma Group [22] analyzed university medical centers plus those ones treating more than 10 RPS per year in comparison to centers following less than 10 RPS per year, finding relevant differences regarding tumor biopsy policy, resection strategies and multimodal therapies. Only 11 surgical departments on 191 surveyed treated more than 10 RPS patients per year; in only 19 hospitals a multidisciplinary sarcoma board was active and in 54% of the departments pretreatment tumor biopsy was a standard procedure. These results suggest the need for dedicated RPS education programs and centralized registration for RPS treatment.

Berger et al. [23] identified 2762 patients from the US National Cancer Database treated for retroperitoneal sarcoma. The majority (59.4%, n = 1642) underwent resection at an academic cancer center. Resection for retroperitoneal sarcoma performed at academic cancer center was an independent predictor of margin-negative resection but was not a statistically significant risk factor for survival, suggesting that site of care may contribute to the quality of retroperitoneal sarcoma surgery.

The management of soft tissue sarcomas requires integrated care at a referral center, as suggested by existing guidelines and consensus statements. Diagnosis of the primary lesion, distant metastasis, or subsequent local recurrence require the use of advanced imaging as well as the expertise of appropriately trained teams. Experts involved in soft tissue sarcoma care suggest treatment with respect to using, dosing, and timing of radiation and chemotherapy tailored for every individual patient [5, 24].

Surgery of RPS, especially for wide re-excision after unplanned primary excision of a mass, requires specific multidisciplinary teamwork [25, 26]. There are data concerning RPS which show that patients treated in sarcoma reference centers can achieve better oncological outcomes [17, 18].

In this study, we collected data concerning the treatment of RPS from 22 hospitals, of which 20 (90%) treated less than 5 cases per year; the low quality of retrieved information from this low volume activity hospitals (LVSCH), mirrors the incidental character of this type of surgery.

We considered margins as macroscopically negative (R0/R1) or macroscopically positive (R2), as available literature shows that this margin classification has a definite prognostic value without great differences between R0 and R1 in the retrospective setting; is often difficult to correctly assess microscopic margins in big retroperitoneal masses in absence of a real compartment or of the possibility of a wide excision [6, 12, 13, 27–30]. The multivariate analysis confirmed that, within high CCV centers, the one with a dedicated surgical team and a RMB (HVCCC) had a better quality of macroscopic margins and a higher rate of intact tumor resection. Keung et al. [31] highlights the importance of maintaining tumor specimen integrity during surgery because tumor fragmentation is independently associated with worse PFS and OS. Bonvalot and colleagues [32] similarly reported that tumor rupture was associated with worse OS. Maintaining tumor specimen integrity is often a daunting challenge given the large size, location, and adjacent organ involvement of many of these tumors, and therefore, tumor integrity can be considered a proxy of surgical quality.

It is expected that outcomes directly under the surgeon’s control, that is, the completeness of resection, are more strongly associated with surgeon volume, a marker of surgical expertise, rather than by hospital volume, which is a somewhat imprecise marker of surgeon experience as well as hospital structure and process characteristics.

Important limitations of this study are its retrospective nature, based on histopathological reports, the omission of non-surgically treated patients, the retrieval of missing data from different databases and the absence of clinical history and follow-up information, particularly about RFS, in patients treated in LVSCH.

## Conclusion

Outside reference or tertiary care centers, the quality of RPS management may be lower because the relevance of both tumor integrity and surgical margin quality are not completely understood and therefore, documented.

In light of the persistent association between improved surgical oncology outcomes and high-volume activity, the centralization of high-risk cancer surgery has been proposed [26–28]. A volume-outcome relationship exists for RPS so, centralization may improve outcomes for RPS keeping in mind that surgical experience plays a larger role for these outcomes than structural/process characteristics.
